# SET mediates TCE-induced liver cell apoptosis through dephosphorylation and upregulation of nucleolin

**DOI:** 10.18632/oncotarget.16785

**Published:** 2017-04-03

**Authors:** Xiaohu Ren, Xinfeng Huang, Xifei Yang, Yungang Liu, Wei Liu, Haiyan Huang, Desheng Wu, Fei Zou, Jianjun Liu

**Affiliations:** ^1^ Key Laboratory of Modern Toxicology of Shenzhen, Shenzhen Center for Disease Control and Prevention, Shenzhen, 518055, China; ^2^ School of Public Health, Southern Medical University, Guangzhou, 510515, China

**Keywords:** trichloroethylene, nucleolin, SET, hepatic cytotoxicity, proteomics

## Abstract

Trichloroethylene (TCE) is an occupational and environmental chemical that can cause severe hepatotoxicity. While our previous studies showed that the phosphatase inhibitor SET is a key mediator of TCE-induced liver cell apoptosis, the molecular mechanisms remain elusive. Using quantitative phosphoproteomic analysis, we report here that nucleolin is a SET-regulated phosphoprotein in human liver HL-7702 cells. Functional analysis suggested that SET promoted dephosphorylation of nucleolin, decreased its binding to its transcriptional activator, c-myc, and upregulated nucleolin expression in TCE-treated cells. Importantly, TCE-induced hepatocyte apoptosis was significantly attenuated when nucleolin was downregulated with specific siRNAs. These findings indicate that TCE may induce hepatocyte apoptosis via SET-mediated dephosphorylation and overexpression of nucleolin.

## INTRODUCTION

Trichloroethylene (TCE) is a chlorinated hydrocarbon that has long been used as an industrial solvent. Because of inappropriate handling and transportation, TCE has become a major environmental contaminant. Both the U.S. Environmental Protection Agency (EPA) and the International Agency for Research on Cancer (IARC) have characterized TCE as a human carcinogen. TCE is mainly metabolized in the liver and causes hepatic toxicity by altering the status of cytochrome P450 [1]. Occupational exposure to TCE has been linked to severe liver dysfunction [2], and epidemiological studies suggested a strong association of TCE exposure with liver cancer [3, 4]. In animal studies, long-term oral gavage with TCE induced hepatocellular proliferation, mitochondrial dysfunction, and glycogen depletion [5]. We have previously identified SET (I2PP2A) also known as protein phosphatase type 2A inhibitor II [6] as a key mediator of TCE-induced liver cell apoptosis [7, 8]. However, whether SET-mediated changes in protein phosphorylation patterns are involved in TCE-induced cytotoxicity remains unknown. Using quantitative phosphoproteomic methods, we characterized SET-related protein phosphorylation profiles and found that dephosphorylation and upregulation of nucleolin was crucial for TCE-induced hepatic cell apoptosis.

## RESULTS

### SET knockdown alters nucleolin phosphorylation status in TCE-treated cells

To assess the influence of SET on protein phosphorylation changes induced by TCE, we characterized protein phosphorylation by mass spectrometry in control and SET-siRNA-transfected (SET-knockdown) L-02 cells. After treatment with TCE, 26 phosphosites on 16 proteins were found to be altered in SET-competent, control L-02 cells, whereas 15 phosphosites on 12 proteins showed differential changes in SET-knockdown L-02 cells (Table 1 and Table 2). Among these changes, the phosphorylation of nucleolin residues S184 and S206 was decreased in control cells, and increased instead after SET-knockdown.

**Table 1:** Identification of TCE induced abnormal (de)-phosphorylation of 27 sites in 16 proteins

Uniport accessions	Protein descriptions	Fold change#	p value	Phosphorylated peptides*	phosphorylated sites
Q9UQ35	Serine/arginine repetitive matrix protein 2	-1.214	0.036429	RGEGDAPFSEPGTT STQRPSpSPETATK	S323
Q9UQ35	Serine/arginine repetitive matrix protein 2	-1.74236	0.02238	SATRPpSpPSPER	S351 S353
Q9UQ35	Serine/arginine repetitive matrix protein 2	-1.28055	0.040114	SLpTpRSPPAIR	T2069 S2071
P46821	Microtubule-associated protein 1B	-1.49788	0.010365	ESpSPLpYSPTFSDSTSAVK	S1793 S1797
P19338	Nucleolin	-1.85542	0.005201	AAAAAPApSEDEDDED DEDDEDDDDDEEDDpS EEEAMETTPAK	S184 S206
P49736	DNA replication licensing factor MCM2	-1.39098	0.004499	GLLYDpSDEEDEERPAR	S139
O00193	Small acidic protein	-1.81218	0.036586	RSApSPDDDLGSSNWEAA DLGNEER	S17
Q15149-4	Isoform 4 of Plectin	-3.62735	0.029429	TSpSEDNLYLAVLR	S21
Q9UHB6	LIM domain and actin-binding protein 1	-2.06584	0.018827	QQpSPQEPK	S698
Q9UHB6	LIM domain and actin-binding protein 1	-5.05972	0.000401	QQpSPQEPK	S698
Q8NE71	ATP-binding cassette sub-family F member 1	-2.06663	0.007659	KAEQGpSEEEGEGEEEEE EGGESK	S228
Q8NE71	ATP-binding cassette sub-family F member 1	-1.83936	0.009453	QQPPEPEWIGDGESTpSPSDK	S22
Q8WX93	Palladin	-1.94712	0.012633	IApSDEEIQGTK	S893
P55081	Microfibrillar-associated protein 1	1.496243	0.009119	IVEPEVVGEpSpDSEVEGDAWR	S116 S118
O95218	Zinc finger Ran-binding domain-containing protein 2	-2.58179	0.019206	ENVEYIEREEpSDGEYDEFGR	S120
Q13541	Eukaryotic translation initiation factor 4E-binding protein 1	1.054209	0.008982	RVVLGDGVQLPPGDYpST TPGGTLFSpTTPGGTR	S37 T46
Q13442	28 kDa heat- and acid-stable phosphoprotein	-1.36106	0.012011	SLDpSDpESEDEEDDYQQK	S60 S63
Q92688	Acidic leucine-rich nuclear phosphoprotein 32 family member B	-2.38103	0.04378	KREpTDDEGEDD	T244
P11137	Microtubule-associated protein 2	1.324371	0.032474	VDHGAEIITQpSPGR	S1782
Q9H3N1	Thioredoxin-related transmembrane protein 1	-2.01394	0.049445	KVEEEQEADEEDVpSEEEAESK	S247

# positive fold changes indicate up-regulation and negative fold changes indicate down-regulation.

* the amino acid letters after the lower case of “p” indicate phosphorylation sites.

**Table 2:** Identification of SET mediated abnormal (de)-phosphorylation of 15 sites in 12 proteins in TCE treated L-02 cells

Uniport accessions	Protein descriptions	Fold change#	p value	Phosphorylated peptides*	phosphorylated animo acids
**P07900-2**	Isoform 2 of Heat shock protein HSP 90-alpha	1.512643	0.010983	ESEDKPEIEDVGpSDEEEEK	S385
**P19338**	Nucleolin	3.542418	0.005201	AAAAAPApSEDEDDEDDEDDE DDDDDEEDDpSEEEAMETTPAK	S184 S206
**Q15149-4**	Isoform 4 of Plectin	-1.22808	0.029429	TSpSEDNLYLAVLR	S21
**Q14247-2**	Isoform 2 of Src substrate cortactin	1.310994	0.034449	TQpTPPVSPAPQPTEER	T364
**Q9UHB6**	LIM domain and actin-binding protein 1	1.908012	0.018827	QQpSPQEPK	S698
**Q8NE71**	ATP-binding cassette sub-family F member 1	1.234261	0.007659	KAEQGpSEEEGEGE EEEEEGGESK	S228
**Q8WX93**	Palladin	2.832776	0.012633	IApSDEEIQGTK	S893
**E9PRY8**	Elongation factor 1-delta	2.171604	0.041186	KPATPAEDDEDDDIDL FGpSDNEEEDKEAAQLR	S578
**Q13541**	Eukaryotic translation initiation factor 4E-binding protein 1	1.52283	0.008982	RVVLGDGVQLPPGDYp STTPGGTLFSpTTPGGTR	S37 T46
**Q13442**	28 kDa heat- and acid-stable phosphoprotein	2.686815	0.012011	SLDpSDpESEDEEDDYQQK	S60 S63
**Q92688**	Acidic leucine-rich nuclear phosphoprotein 32 family member B	2.008763	0.04378	KREpTDDEGEDD	T244
**O94804**	Serine/threonine-protein kinase 10	1.42668	0.039742	QVAEQGGDLpSPAANR	S438

* the lower case of letter “p” before the amino acid represents the position of the phosphorylation and the amino acid is which to have been phosphorylated.

# The fold change data were log2 transformed, thus negative numbers indicated decreasing and positive numbers indicated increasing of phosphorylation.

**Figure 1:**
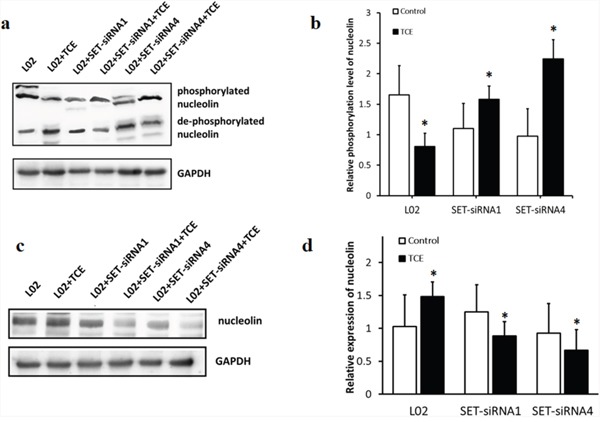
SET decreases phosphorylation of nucleolin and increases its expression in TCE-exposed liver cells L-02 cells were interfered with two different SET-siRNA oligonucleotides. **(a)** phosphorylated and unphosphorylated nucleolin were separated by SDS-PAGE; **(b)** after TCE treatment, the phosphorylation of nucleolin decreased in control L-02 cells and increased after SET-siRNA-transfection (*: *P* < 0.05); **(c)** nucleolin expression, protein band image; **(d)** after TCE treatment, the expression of nucleolin increased in control L-02 cells and decreased after SET-siRNA-transfection (*: *P* < 0.05, compared with control). Note that the pattern of nucleolin expression was inversely correlated with its pattern of phosphorylation.

To confirm the above results, lysates from SET-siRNA-transfected L-02 cells (SET-siRNA1 and SET-siRNA4) were analyzed by phosphate-affinity SDS-PAGE. The presence of Phos-Tag™ in the acrylamide gels reduces the mobility of phosphorylated proteins, allowing further separation of differentially phosphorylated proteins. The results indicated that following TCE treatment, nucleolin was dephosphorylated and upregulated in L-02 cells, while knockdown of SET prevented these changes (Figure 1).

### Nucleolin dephosphorylation prevents its binding to c-myc

**Figure 2:**
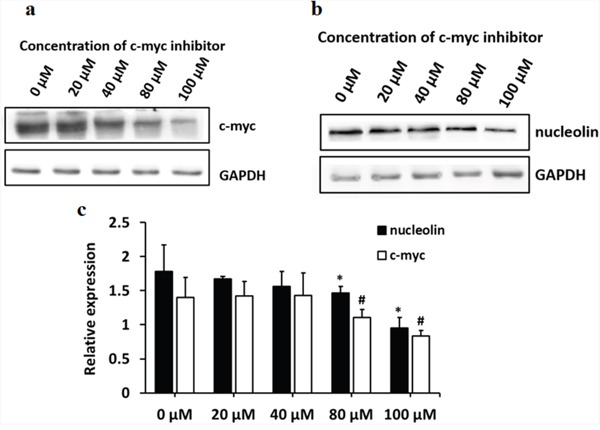
c-myc controls the expression of nucleolin in L-02 cells L-02 cells were treated with different concentrations of 10058-F4, a c-myc inhibitor. **(a)** c-myc protein band; **(b)** nucleolin protein band; **(c)** both c-myc and nucleolin expression were significantly decreased in cells treated with c-myc inhibitor concentrations of 80 μM and 100 μM (#: *P* < 0.05, relative expression of c-myc, compared with 0 μM; (*: *P* < 0.05, relative expression of nucleolin, compared with 0 μM).

**Figure 3:**
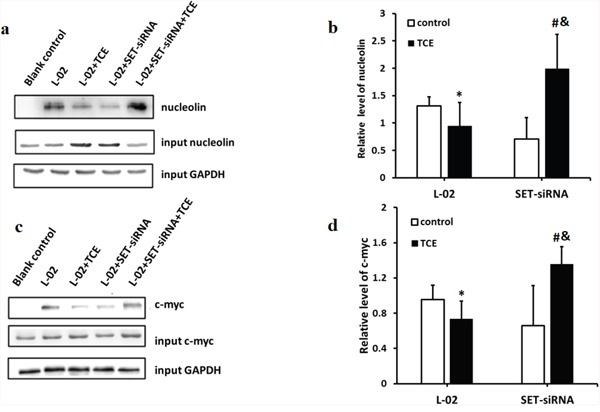
Dephosphorylation impairs the ability of nucleolin to bind to c-myc **(a)** c-myc protein band eluted from immobilized nucleolin; **(b)** the ability of c-myc to capture nucleolin was impaired in TCE-treated L-02 liver cells, but improved however after SET knockdown (*: *P* < 0.05, compared with control; &: *P* < 0.05, compared with TCE-treated L-02 cells); **(c)** nucleolin protein band eluted from immobilized c-myc; **(d)** the ability of nucleolin to capture c-myc was impaired by TCE treatment in L-02 cells; this interaction improved after SET knockdown (*: *P* < 0.05, compared with control; &: *P* < 0.05, compared with TCE treated, L-02 control cells).

C-myc was reported to be a major transcriptional regulator of nucleolin, while nucleolin, in turn, negatively regulates the transcription of c-myc [9, 10]. Since the overall phosphorylation status of nucleolin was found to be decreased in TCE-treated L-02 liver cells, and this was prevented by SET knockdown, co-IP experiments were performed to determine whether the phosphorylation of nucleolin affected its binding to c-myc. Results indicated that inhibition of c-myc downregulated nucleolin expression (Figure 2), and TCE-induced nucleolin dephosphorylation inhibited in turn its binding to c-myc (Figure 3).

### Nucleolin knockdown attenuates SET-mediated hepatic cell apoptosis induced by TCE exposure

**Figure 4:**
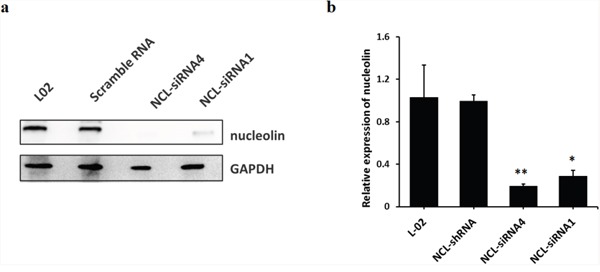
Stable knockdown of nucleolin in L-02 cells **(a)** nucleolin protein band; **(b)** successful nucleolin knock down in L-02 cells by lentivirus-mediated RNA interference. The efficacy of NCL-siRNA4 was higher than that of NCL-siRNA1 (*: *P* < 0.05, compared with L-02 control cells; **: *P* < 0.01, compared with L-02 control cells).

**Figure 5:**
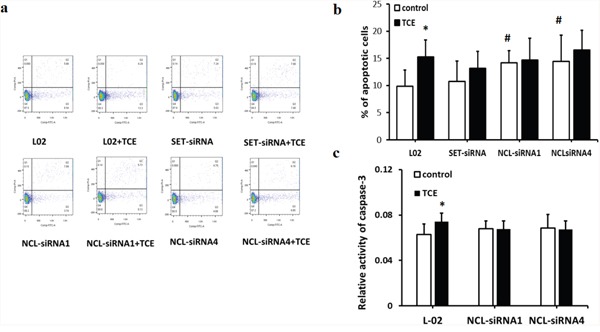
SET and nucleolin siRNAs attenuate TCE-induced apoptosis in human liver cells **(a)** flow cytometry analysis of cell apoptosis by PI/Annexin-V staining; **(b, c)** TCE-induced apoptosis in L-02 cells was attenuated after SET knockdown and nucleolin knockdown (*: *P* < 0.05, compared with control,; #: *P* < 0.05, compared with L-02 cells); **(c)** caspase-3 activity assay indicates inhibition of apoptosis upon nucleolin knockdown in TCE-treated L-02 cells (*P* < 0.05).

To test the hypothesis that SET-mediated nucleolin overexpression contributes to TCE-induced apoptosis, L-02 cells were transfected with lentivirus-containing siRNAs against nucleolin. Western-blot analysis indicated that nucleolin was successfully down-regulated in L-02 cells (Figure 4). TCE-induced apoptosis of L-02 cells was suppressed after SET and nucleolin knockdown (Figure 5). These data suggest that nucleolin expression contributes to SET-mediated apoptosis in liver cells exposed to TCE.

## DISCUSSION

**Figure 6:**
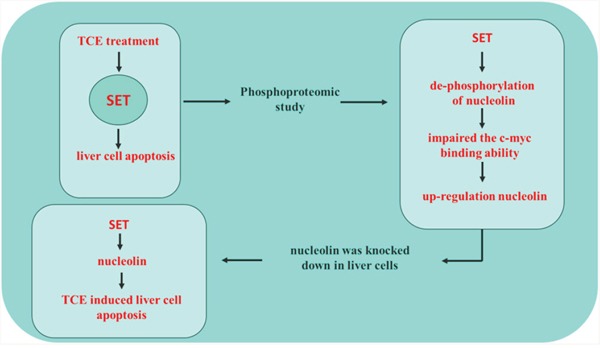
SET mediates TCE-induced liver cell apoptosis through upregulation of nucleolin After TCE exposure, SET up-regulates nucleolin by promoting its dephosphorylation, which impairs its ability to bind its transcriptional activator, c-myc, further leading to liver cell toxicity.

SET is amulti-functional protein that specifically inhibits phosphatase 2A in eukaryotic cells, and has been linked to liver carcinogenesis [11]. Our previous studies suggested that SET is as a key mediator of TCE-induced apoptosis in hepatocytes, although the specific mechanisms remained unclear [8, 12, 13]. Using phosphoproteomics analysis, we found that the phosphorylation status of nucleolin was differently affected (increased) upon siRNA-mediated SET knockdown in TCE-exposed human liver L-02 cells. We also found that nucleolin phosphorylation was negatively associated with its own expression. Our data showed that dephosphorylation of nucleolin impaired its ability to bind c-myc, a major transcription factor that induces the expression of many genes, including nucleolin. Since in its phosphorylated state nucleolin represses c-myc activity, dephosphorylation of nucleolin resulted in an increase in its own expression. Furthermore, our study underscored a major role of nucleolin in TCE-mediated liver cell toxicity, as nucleolin knockdown attenuated L-02 cell apoptosis following treatment with TCE (Figure 6). Thus, our results identify nucleolin as a SET-regulated phosphoprotein critically involved in TCE-induced liver cell toxicity.

Nucleolin is an abundant, multifunctional phosphoprotein mainly located in the nucleolus. It is involved in chromatin structure stabilization, rRNA maturation, ribosome assembly, and nucleo-cytoplasmic transport [14]. Cell division cycle 2 kinase (cdc2)-mediated phosphorylation of nucleolin was found to be important for cell mitosis [15, 16]. A potential contribution of nucleolin to carcinogenesis has been highlighted by past investigations. For instance, nucleolin was shown to promote transformation of non-small cell lung cancer by regulating cytosolic phospholipase 2A [17]. Another study showed that phosphorylation-induced redistribution of nucleolin could promote tumor metastasis through the PI3K/Akt pathway in colorectal carcinoma [18]. Nucleolin has also been observed to suppress Fas-mediated apoptosis by blocking the binding of Fas ligands to Fas [19]. Of potential therapeutic interest, a small amount of nucleolin has been found to be expressed on cell membranes, serving as a receptor for PEGylated, poly-lysine packaged DNA particles [20].

In summary, the present study demonstrated that SET-mediated dephosphorylation of nucleolin in TCE-treated human L-02 liver cells promotes nucleolin expression by suppressing its c-myc binding ability. Importantly, SET and nucleolin knockdown prevented TCE-induced apoptosis in these cells. Although further work is needed to characterize in detail the mechanisms underlying these observations, the present findings provide important insights into the molecular bases of TCE hepatotoxicity, and will hopefully help guide prevention and treatment efforts.

## MATERIALS AND METHODS

### Reagents

The human liver HL-7702 (L-02) cell line was purchased from the Institute of Cell Biology of the Chinese Academy of Sciences (Shanghai, China). SET-siRNA transfected L-02 cells were derived from a previous study [8]. The iTRAQ 8 Plex Labeling Kit was purchased from SCIEX (Framingham, MA, U.S.). Reagents for cell culture, titanium dioxide spin columns, Co-IP kit, Alexa Fluor® 488 Annexin V/Dead Cell Apoptosis Kit, C18 columns, and electro-generated chemical luminescence substrates were obtained from Thermo Scientific (Rockford, IL, U.S.). Sequencing grade trypsin, triethylammonium bicarbonate (TEAB), acetonitrile, isopropyl alcohol, pyrroline, and ammonium hydroxide were obtained from Sigma-Aldrich (Shanghai, China). The Phos-Tag™ reagent was purchased from Wako Pure Chemicals (Richmond, VA, U.S.). Antibodies against nucleolin and c-myc were purchased from Abcam (Cambridge, MA, U.S.). Antibody against GAPDH were purchased from Santa Cruz Biotechnology (Dallas, TX, U.S.). Reagents for Western-blot were obtained from Bio-Rad (Hercules, CA, U.S.).

### Cell culture and treatment

L-02 cells and SET-siRNA-transfected L-02 cells were cultured in RPMI-1640 medium supplemented with 12% fetal bovine serum, 100 μg/ml streptomycin and 100 units/ml penicillin at 37°C with 5% CO_2_. The cells were treated with TCE at a concentration of 8 mmol/L, which was chosen based on previous observations [21]. TCE was pre-dissolved in 50 μL of DMSO and added to 10 mL of culture medium. Treatment lasted 24 h. Separately, L-02 cells were treated with different concentrations of a c-myc inhibitor (10058-F4, Santa Cruz) for 24 h.

### Phosphoproteomics study

After treatment with TCE cells were harvested and centrifuged at 1,200 rpm. Proteins were extracted by PhosphoSafe Extraction Reagent (Thermo Scientific). Protein buffer was then replaced by TEAB using 3kDa filtration devices (Millipore). Total protein levels were quantified using a BCA protein quantification kit (Thermo Scientific). Proteins were digested with trypsin and digestion products were labeled with iTRAQ (8-plex); a pooled sample from all groups served as the internal control. The TiO_2_-enriched phosphopeptides were separated using liquid chromatography (3 μm, 100 Å, 75 μm i.d. × 15 cm, Acclaim PepMap100, C18, Dionex) and analyzed using mass spectrometry (TripleTOF 5600 System, AB SCIEX). Data were processed and searched against the Uniprot human protein database (a total of 71,434 entries) with the MASCOT search engine. All reporter ion intensities groups were fractioned to internal standard, and the *limma* package for R 3.1.1 (obtained from Bioconductor, http://bioconductor.org) was used for quantitative analysis of phosphopeptides.

### Western blot analysis

Proteins were extracted for Western blot analysis using PhosphoSafe Extraction Reagent and quantified using a BCA protein quantification kit. Equal amounts (50 μg of each sample) of protein extracts were electrophoresed on Phos-Tag™ / 10% SDS-PAGE gels (containing 0.2 mmol/L MnCl_2_ and 0.1 mmol/L Phos-Tag™). The samples were then transferred onto PVDF membranes and blocked with 5% skimmed milk powder in TBST. Antibodies against nucleolin and c-myc were diluted 1:1,000. Anti–GAPDH antibody was diluted 1:3,000 in TBST (0.2% Tween-20 in TBS). The membranes were incubated with diluted antibodies for 1 h at room temperature, washed 3 times with TBST, and incubated with HRP-conjugated goat anti-mouse antibodies (diluted 1:3,000 in TBST) for 1 h at room temperature. After exposure to ECL substrate, protein band images were captured by ImageQuant RT ECL System (GE Healthcare) and analyzed using ImageQuant TL-1D Analysis Tool. Overall phosphorylation levels were calculated as the ratio between the sum of phosphorylated isoforms and total proteins.

### Co-immunoprecipitation assay

First, 10 μg of antibodies against c-myc and nucleolin were conjugated and cross-linked to protein A/G plus agarose (Pierce Crosslink IP Kit, Thermo). Proteins were extracted from TCE-treated cells in accordance with the manufacturer's instructions and incubated with cross-linked antibodies.

### Recombination of lentiviral vectors and generation of stable nucleolin knockdown cells

Four siRNA oligonucleotides targeting the mRNA of nucleolin (NCL-siRNA), as well as a scrambled RNA oligonucleotide, were designed and synthesized with BamHI and EcoRI restriction site enzymes (Sangon Biotech Co., Ltd.; Shanghai, China). The two siRNAs with the highest interference performance were used in subsequent studies (NCL- siRNA1: gatccGCGATGAGGATGACGAAGATTTCAAGAGAATCTTCGTCATCCTC ATCGTTTTTT, NCL-siRNA4: gatccGGAAGACGGTGAAATTGATTTCAAGAGAATCAATTTCACCGTCTTCC TTTTTT). The siRNAs and scrambled RNA oligonucleotides were cloned into pLVX-shRNA1 plasmids. The pseudotyped lentiviruses were produced by co-transfecting recombinant siRNA plasmids with Clontech Lenti-X HT packaging vectors into 293T cells. After 48 h, the lentivirus-containing supernatants were collected by centrifugation at 1,200 rpm for 10 min. L-02 cells were treated with supernatants for 24 h and stable nucleolin knockdown cells were isolated after 2 weeks of selection with puromycin (2 μg/mL). Knockdown efficiency was verified by Western blot analysis. SET-siRNA production and RNA interference efficacy have been described and validated in our previous studies (Liu et al. 2007; Yang et al. 2012).

### Apoptosis assays

### PI/Annexin V- staining assay

After TCE treatment, approximately 5 × 10^5^ of control and NCL-siRNA-transfected liver cells were collected using low speed centrifugation (1,200 rpm for 5 min at room temperature). The cells were washed twice with PBS, resuspended in the Alexa Fluor® 488 Annexin V binding buffer and then stained with PI/ Alexa Fluor 488Annexin V-at room temperature in the dark for 15 min. Apoptosis levels were measured using a flow cytometer (BD FACSCanto™ II).

### Caspase-3 activity assay

Caspase-3 activity was measured using a Caspase-3 activity kit in accordance with the manufacturer's instructions (Biovision). Cells were collected and washed 3 times in ice-cold PBS, re-suspended in lysis buffer, incubated on ice for 20 min, and then centrifuged at 12,000g at 4°C for 10 min. The supernatant was kept and protein concentration was quantified using a BCA protein quantification kit (Thermo). Then 100 μg of protein extracts were diluted with dilution buffer and incubated with DEVD-pNA substrate at 37°C for 1 h. The samples’ absorbance was measured at 400 nm using a TECAN Infinite M1000 microplate reader.

## SUPPLEMENTARY MATERIALS DATA


