# Supplementary material for: SET mediates TCE-induced liver cell apoptosis through dephosphorylation and upregulation of nucleolin
Source: Oncotarget. 2017 Apr 3;8(25):40958–66. doi: 10.18632/oncotarget.16785 (PMC5522280; doi:10.18632/oncotarget.16785)
Supplement: Supplementary file 1 [file oncotarget-08-40958-s001.pdf]

SET mediates TCE-induced liver cell apoptosis through dephosphorylation and upregulation of nucleolin

SUPPLEMENTARY DATA

Figure 1b: the overall phosphorylation of nucleolin was decreased in TCE treated liver cells (0.49 ± 0.13), however increased in TCE treated liver cells with SET knockdown (SET-siRNA1: 1.44 ± 0.20, SET-siRNA2: 2.30 ± 0.32).

Figure 1d: NCL was up-regulated in TCE treated L-02 cells (1.44 ± 0.62), however down-regulated in TCE treated L-02 cells with SET knockdown (SET-siRNA1: 0.71 ± 0.28, SET-siRNA2: 0.72 ± 0.20).

Figure 2c: c-myc was decreased in the presence of c-myc inhibitor at 80 μM (0.79 ± 0.07) and 100 μM (0.60 ± 0.11) in L-02 cells.

Nucleolin was decreased in the presence of c-myc inhibitor at 80 μM (0.82 ± 0.07) and 100 μM (0.53 ± 0.04) in L-02 cells.

Figure 3b: The ability of c-myc to capture nucleolin was impaired in TCE treated L-02 cells (0.72 ± 0.30), however improved in TCE treated cells with SET knockdown (2.83 ± 0.89).

Figure 3d: The ability of nucleolin to capture c-myc was impaired in TCE treated L-02 cells (0.77 ± 0.20), however improved in TCE treated liver cells with SET knockdown (2.05 ± 0.31).

Figure 4b: Nucleolin was knocked down by two NCL-siRNA nucleotides. The inhibitory efficacy of NCL-siRNA4 (0.19 ± 0.02) was higher than NCL-siRNA1 (0.28 ± 0.05).
